# *ITGB4* upregulation is associated with progression of lower grade glioma

**DOI:** 10.1038/s41598-023-49801-y

**Published:** 2024-01-03

**Authors:** Pengyu Chen, Tuo Ma, Tianfang Yan, Zhenhua Song, Chengyong Liu, Chao Pan, Baoshuang Zhang, Danian Wei, Guohui Wang

**Affiliations:** 1 Department of Treatment Center For Traumatic Injuries, The Third Affiliated Hospital of Southern Medical University, Guangzhou, 510630 China; 2 Department of Nuclear Medicine, The Fourth Hospital of Hebei Medical University, Shijiazhuang, Hebei China; 3 Department of Neurological Diagnosis and Restoration, Osaka University Graduate School of Medicine, Osaka, Japan; 4 Department of Radiotherapy, Tianjin First Center Hospital, Tianjin, China

**Keywords:** Cancer, CNS cancer

## Abstract

Gliomas originating in the neuroepithelium account for about 80% of brain malignancies and are the most common cancer of the central nervous system. Clinical management of gliomas remains challenging despite significant advances in comprehensive therapies, including radiotherapy, chemotherapy, and surgery. The *ITGB4* (Integrin subunit beta 4) gene encodes a receptor for laminins and its upregulation in tumor tissues is associated with poor prognosis. However, its role in glioma is not well understood. First, we performed a pan cancer analysis of *ITGB4* expression in The Cancer Genome Atlas (TCGA) dataset. Survival analysis was done on Chinese Glioma Genome Atlas (CGGA) and TCGA. Immunohistochemistry was then used to validate the expression and role of *ITGB4* in glioma. We finally analyzed the possible mechanism by immune infiltration and single-cell sequencing analysis. Here, we found that *ITGB4* is upregulated in glioma and accurately predicts the prognosis of lower grade glioma (LGG). Univariate and multivariate Cox regression analyses showed that *ITGB4* is a risk factor for LGG. Immunohistochemical analysis confirmed that *ITGB4* accurately predicts LGG prognosis. Non-negative matrix factorization (NMF) cluster analysis showed that *ITGB4* was closely related to immune related genes. Immune cell infiltration and single cell sequencing analyses indicated that *ITGB4* may be closely related to the microenvironment of gliomas, especially tumor-associated fibroblasts. *ITGB4* is a promising diagnostic and therapeutic factor in LGG patients.

## Introduction

Gliomas originating in the neuroepithelium account for about 80% of brain malignancies and are the commonest cancer type of the central nervous system (CNS)^1^. Based on the degree of malignancy, the WHO classifies glioma into grade 1–4^2^, with grade 2, 3, and 4 associated with relatively poor prognoses due to infiltrative growth, difficulty of surgical removal, and resistance to chemoradiotherapy^3^. In recent years, the diagnosis and evaluation of glioma have changed greatly, and include a combination of histopathology and molecular markers. The 5^th^ edition of the WHO classification of tumors of the CNS emphasizes the importance of molecular and integrated diagnostics in diagnosing and treating glioma^4^. Although tumor electric field therapy is one of the most promising treatment strategies, in glioma it is associated with a low 5-year survival rate. After treatment, some LGGs recur and progress into high-grade gliomas. Thus, effective biomarkers for the treatment of glioma are urgently needed.

Integrins are heterodimeric transmembrane receptors composed of α and β subunits and are made of 24 different proteins. They regulate various biological functions, including tumor proliferation, migration, and invasion. *ITGB4* gene, a member of the integrin family, encodes the integrin β4 subunit and mediates cell adhesion or extracellular matrix by interacting with fibronectin and collagen and also acts as a receptor for laminin^5^. *ITGB4* has been implicated in various cancers, including prostate cancer, colorectal cancer, and lung cancer and may correlate with poor prognosis^6–8^. It is reported to mediate prostate cancer cells mobility and invasion in vitro^6^, and to promote colon cancer progression by regulating cell proliferation, migration, and apoptosis, indicating that it may have therapeutic and prognostic value in colon cancer^7^. *ITGB4* promotes pancreatic cancer progression by regulating MEK-ERK signaling, which is closely associated with prognosis^9^. However, few studies have investigated the role of *ITGB4* in gliomas, especially LGGs.

Here, we analyzed *ITGB4* expression in glioma and its association with glioma prognosis on TCGA and CGGA. We also analyzed its possible mechanism through immune infiltration and single cell sequencing. Finally, the expression of *ITGB4* in glioma and its relationship with overall survival (OS) were analyzed using immunohistochemistry.

## Materials and methods

### Data acquisition and processing

RNAseq data and associated clinical data from patients with LGG and glioblastomas (GBM) were downloaded from TCGA (https://portal.gdc.cancer.gov/repository). Chinese gene expression profiles and corresponding clinical data from glioma were obtained from CGGA (http://www.cgga.org.cn/download.jsp). The RNAseq data from TCGA and CGGA were standardized and batched using limma package on R. Patients with unknown or incomplete clinicopathological parameters were excluded from the analysis. Single cell RNAseq data were obtained from dataset GSE117891^10^ (https://www.ncbi.nlm.nih.gov/geo/query/acc.cgi?acc=GSE117891).

### *ITGB4* differential expression analysis

First, we performed a pan cancer analysis of *ITGB4* expression. RNAseq data on 33 tumor tissues were downloaded from TCGA and data on corresponding normal tissues downloaded from The Genotype-Tissue Expression (GETx). Wilcoxon rank sum test was used to compare *ITGB4* expression in tumor tissues vs corresponding normal tissues.

### Prognostic analysis

The TCGA data on the 33 tumors were subjected to univariate cox regression analysis to determine the effects of *ITGB4* on OS, progression free interval (PFI) and disease specific survival (DSS). Next, patients with glioma (LGG and GBM) were grouped into the ‘high’ or ‘low’ groups based on median value of *ITGB4* expression. Patients with LGG were stratified based on clinicopathological features, including age (≤ 40, > 40), gender (female/male), grade (Grade 2, and Grade 3), IDH status (mutation, wildtype), 1p19q status (codel, non-codel), and MGMT (methylated, unmethylated). Kaplan–Meier analysis was used to determine survival in the low and high *ITGB4*-expression groups. Univariate and multivariate Cox regression analysis was used to assess if *ITGB4* is an independent prognostic factor for patients with LGG.

### Immunohistochemical staining and scoring

Glioma tissue microarray services were purchased to Shanghai Outdo Biotech Co., Ltd., China. Glioma was diagnosed by pathology. *ITGB4* was detected using monoclonal antibody (14803, CST). Human tissues were stained using mouse and rabbit specific HRP/DAB detection IHC Kit (ab64264, Abcam) according to the manufacturer’s instructions. Immunohistochemical scoring was done as follows based on previously published articles^11^: 0 = no staining, 1 = weak staining, 2 = moderate staining, and 3 = strong staining. The positive staining rate was scored as follows: 0 (0–5%), 1 (5–25%), 2 (26–50%), 3 (51–75%), and 4 (> 75%). A score of < 3 was considered negative and > 3 positive.

### NMF cluster analysis

We performed NMF cluster analysis on CGGA693 database. First, we obtained the immune related genes from the ImmPort database, then extracted the expression of immune related genes from CGGA693 database, and combined with the survival time and survival status of patients to perform NMF clustering.

### Evaluation of the effect of *ITGB4* on glioma microenvironment

The proportion of immunocytes in gliomas was calculated using single-sample gene set enrichment analysis (ssGSEA). Infiltration of immune cells was quantified using enrichment scores calculated with R's GSVA package (gene set variation analysis). In order to analyze the relationship between *ITGB4* expression and TumorPurity, StromalScore, ESTIMATEScore, and ImmuneScore, Spearman correlation analysis was used.

### Database for annotation, visualization and integrated discovery (DAVID)

Our analysis identified differentially expressed genes (DEGs) between glioma patients with high or low expression of *ITGB4* using the limma package on R utilizing |logFC|> 1 and FDR <  = 0.05. DAVID 6.8 (https://david.abcc.ncifcrf.gov/) was used to further explore its possible mechanism.

### Single cell sequencing analysis

Seurat R package was used to reprocess the count matrix in which the dimensional reduction plot and cell type annotation. *ITGB4* expression pattern was visualized using the Feature Plot function.

### Statistical analyses

*ITGB4* levels in tumor vs normal tissues were compared using a Wilcoxon signed-rank test. Spearman’s rank test was used to assess correlation between *ITGB4* expression and TumorPurity, StromalScore, ESTIMATEScore, and ImmuneScore. Kaplan–Meier analysis was used assess the effect of *ITGB4* on prognosis. Statistical analyses were done on SPSS 24.0 (IBM), GraphPad Prism 6, and R 4.0.1. *P* =  < 0.05 was considered statistically significant.

### Ethics approval and consent to participate

The article data is obtained from the network public database in accordance with the database policy. And the informed consent was not required.

## Results

*ITGB4* expression was assessed in publicly available datasets from TCGA and CGGA. There were 587 and 686 cases in TCGA and CGGA, respectively. A tissue microarray dataset from 169 cases was also included in the analyses. Clinical and molecular features are shown on Table 1.

**Table 1:** Clinicopathological characteristics of glioma patients from the TCGA, CGGA database and tissue microarray.

	TCGA (n = 587)	CGGA (n = 686)	Tissue microarray (n = 169)
Age			
≤ 40	241	307	38
> 40	346	379	131
Gender			
Female	246	287	62
Male	341	399	107
Normal tissue	NA	NA	NA
Grade			
2	211	177	97
3	234	226	51
4	142	283	21
IDH status			
Wildtype	219	315	NA
Mutation	368	371	NA
1p/19q			
Codel	149	141	NA
Non-codel	438	545	NA
MGMT			
Methylated	NA	386	NA
Un-methylated	NA	300	NA
Status			
Dead	173	457	57
Alive	414	229	112

### *ITGB4* is upregulated in most cancers

**Figure 1:**
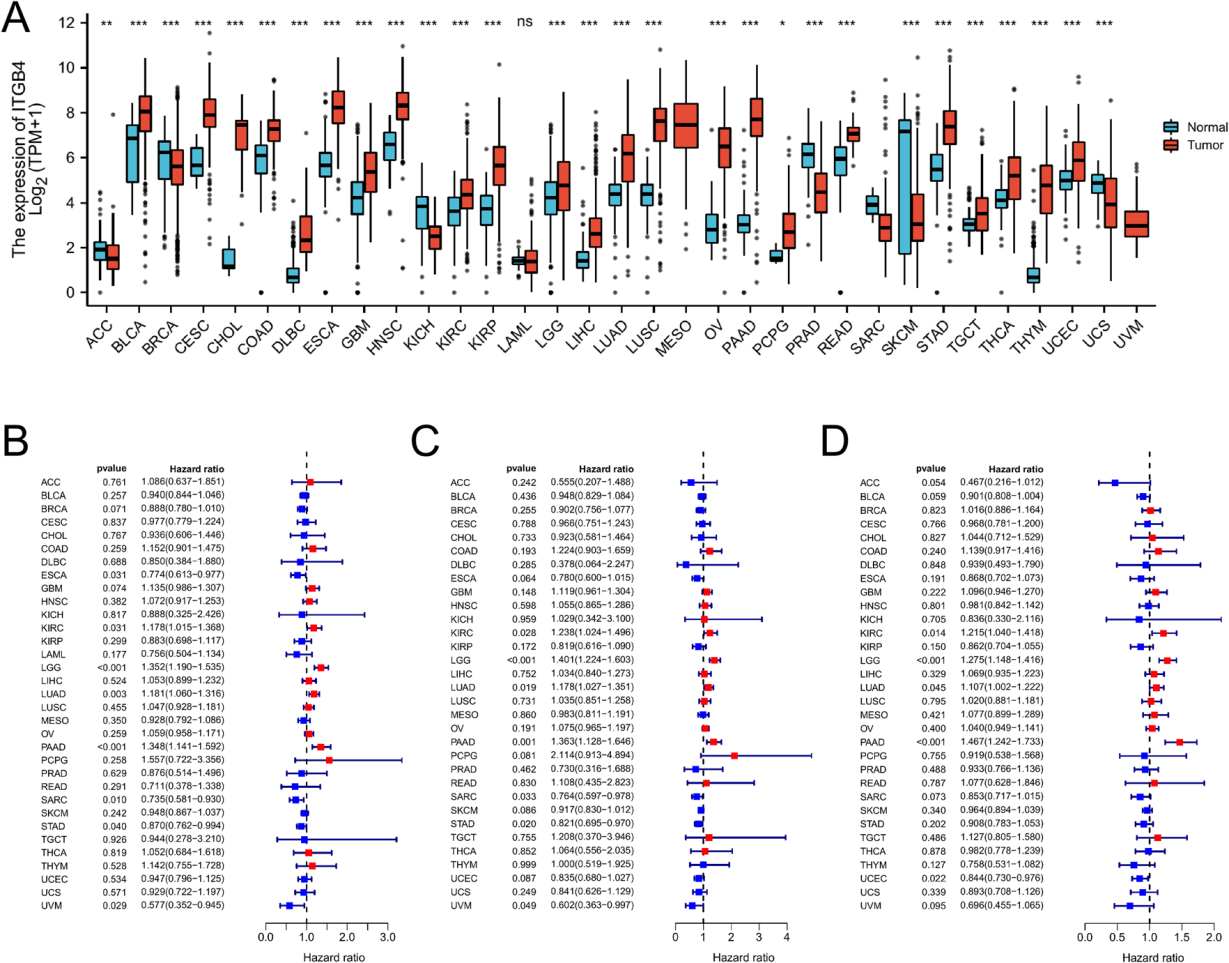
Pan-cancer analysis of *ITGB4* expression. (**A**) analysis of *ITGB4* expression in 33 tumors on TCGA. *, **, and *** indicate *p* =  < 0.05, 0.01, and < 0.001, respectively; ns = not significant (Wilcoxon test). (**B**–**D**) Risk plot of correlation between *ITGB4* levels and OS, PFI, DSS (red represents HR =  > 1 (risky) and blue represents HR =  < 1 (protective).

Pan-cancer analysis of 33 tumors showed that *ITGB4* expression was significantly higher in various cancer types relative to matched normal tissues, including BLCA, CESC, CHOL, COAD, DLBC, GBM, HNSC, LIRC, LGG, LIHC, LUAD, LUSC, OV, PAAD, PCPG, READ, STAD, TGCT, THCA, THYM, and UCEC. In ACC, BRCA, KICH, PRAD, SARC, and SKCM. (Fig. 1A, *, **, and *** indicate *p* =  < 0.05, < 0.01, and < 0.001, respectively, ns = not significant). Abbreviation information could be found in supplementary 1. To investigate the relationship between *ITGB4* expression and OS, PFI, and DSS, patients were divided into the high and low *ITGB4* expression groups. Univariate analysis showed that patients with high *ITGB4* levels had shorter survival (OS, PFI and DSS, Fig. 1B–D). Interestingly, *ITGB4* is a risk factor for LGGs. However, *ITGB4* does not predict GBM prognosis (OS: LGG, HR = 1.352, 95% CI 1.190–1.535, *p* =  < 0.001; GBM: HR = 1.135, 95% CI 0.986–1.307, *p* = 0.074; PFI: LGG, HR = 1.401,95% CI 1.224–1.603, *p* =  < 0.001; GBM: HR = 1.119, 95% CI 0.961–1.304, *p* = 0.148; DSS: LGG, HR = 1.275, 95% CI 1.148–1.416, *p* =  < 0.001; GBM: HR = 1.096, 95% CI 0.946–1.270, *p* = 0.222).

### High *ITGB4* expression correlates with poor prognosis

**Figure 2:**
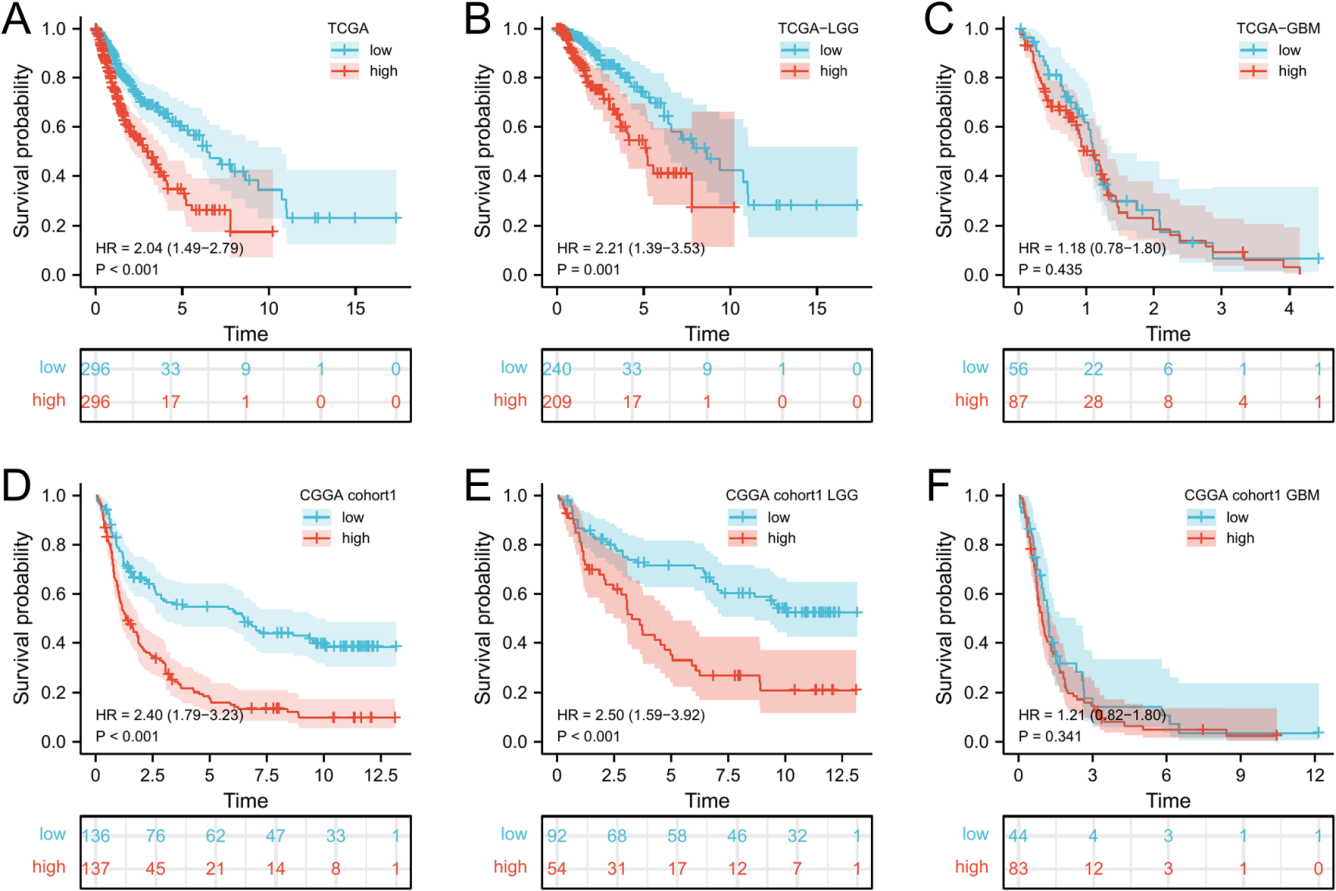
High *ITGB4* levels correlate with poor prognosis in lower grade glioma. (**A**–**C**) Survival analysis revealed that the survival time of patients with high *ITGB4* expression was significantly lower relative to those in the low expression group in (**A**) (TCGA), (**B**) (TCGA-LGG), (**D**) (CGGA), and (**E**) (CGGA-LGG), but there was no significant difference in (**C**) (TCGA-GBM) and (**F**) (CGGA-GBM).

**Figure 3:**
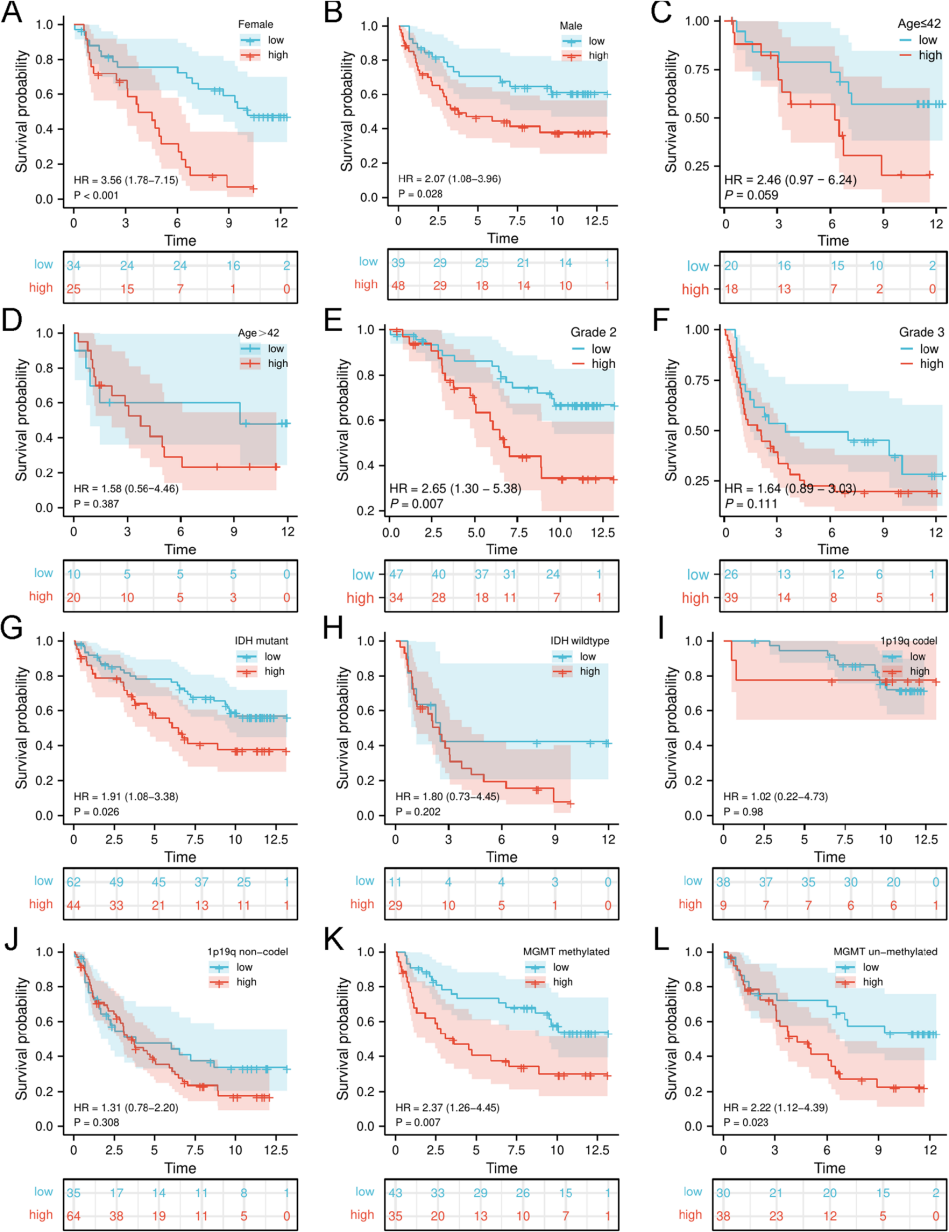
Prediction of outcome of the *ITGB4* in stratified patients in the CGGA dataset. Survival analysis of the signature in patients stratified by gender (**A**,**B**), age (**C**,**D**), grade (**E**,**F**), IDH (**G**,**H**), 1p19q status (**I**,**J**), and MGMT promoter (**K**,**L**).

The results above showed that *ITGB4* is highly expressed in tumors and is an independent prognostic factor in gliomas, especially LGG. Next, we analyzed TCGA and CGGA datasets to determine the impact of *ITGB4* in the survival of LGG and GBM. First, we divided all glioma patients on TCGA into the high and the low expression groups based on median *ITGB4* expression level. This analysis revealed that patients with high *ITGB4* expression had significantly worse OS than those with low expression (HR = 2.04, 95% CI 1.49–2.79, *p* =  < 0.001, Fig. 2A). We then performed a subgroup analysis by dividing LGG patients into the high and low expression groups based on *ITGB4* expression. Survival analysis of this group showed that high *ITGB4* levels correlated with significantly lower OS relative to low *ITGB4* expression (HR = 2.21, 95% CI 1.39–3.53, *p* = 0.001, Fig. 2B). However, this was not the case in GBM in which *ITGB4* was not a prognostic factor (HR = 1.18, 95% CI 0.78–1.80, *p* = 0.435, Fig. 2C). Similar observations were made from the CGGA analysis (glioma: HR = 2.40, 95% CI 1.79–3.23, *p* =  < 0.001; LGG: HR = 2.50, 95%CI 1.59–3.92, *p* =  < 0.001; GBM: HR = 1.21, 95% CI 0.82–1.80, *p* = 0.341, Fig. 2D–F). Our results strongly indicate that *ITGB4* can accurately predict LGG prognosis. Next, stratification analysis was done to evaluate the impact of *ITGB4* in glioma prognosis. Analysis of the LGG subgroups based on age, sex, grade, IDH, 1p19q, and MGMT revealed that high *ITGB4* expression correlated with poor survival in females (HR = 3.56, 95% CI 1.78–7.15, *p* =  < 0.001, Fig. 3A) and males (HR = 2.07, 95% CI 1.08–3.96, *p* =  < 0.001, Fig. 3B). Similar observations were made for patients who were not over forty (HR = 1.83, 95%CI 1.38–2.47, P < 0.001, Fig. 3C) and over forty (HR = 1.58 95% CI 0.56–4.46, *p* = 0.387, Fig. 3D). The survival probability of patients who were classified as WHO glioma grade 2 (HR = 2.65, 95% CI 1.30–5.38, *p* = 0.007) was significantly poor. However, those classified as WHO glioma grade 3 did not reach the considered threshold (Fig. 3E,F). Finally, patients with high *ITGB4* expression had a lower survival probability of reaching the threshold in the IDH mutation subgroup (HR = 1.91, 95% CI 1.08–3.38, *p* = 0.026), MGMT methylated subgroup (HR = 2.37, 95% CI 1.26–4.45, *p* = 0.007), and MGMT unmethylated subgroup (HR = 2.22, 95% CI 1.12–4.39, *p* = 0.023), except the IDH wildtype and 1p19q subgroups (Fig. 3G–L). Univariate and multivariate analysis also showed that *ITGB4* was a poor prognostic factor in lower grade glioma (Table 2).

**Table 2:** Univariate and multivariate analysis for overall survival of lower grade glioma.

	Univariate analysis			Multivariate analysis		
	P	HR	95% confidence interval	P	HR	95% confidence interval
Age	0.005	1.033	1.009–1.057	0.204	1.014	0.992–1.037
Gender	0.231	0.762	0.488–1.189	0.173	0.725	0.457–1.151
Grade	0.000	3.072	1.947–4.847	0.000	3.160	1.886–5.295
IDH	0.000	2.974	1.868–4.733	0.779	1.088	0.602–1.966
1p19q	0.000	5.642	2.957–10.764	0.000	5.689	2.846–11.375
MGMT	0.513	1.159	0.743–1.808	0.616	1.138	0.685–1.888
ITGB4	0.000	2.330	1.837–4.339	0.000	1.989	1.532–3.898

### Immunohistochemistry (IHC) indicates *ITGB4* may contribute to poor prognosis

**Figure 4:**
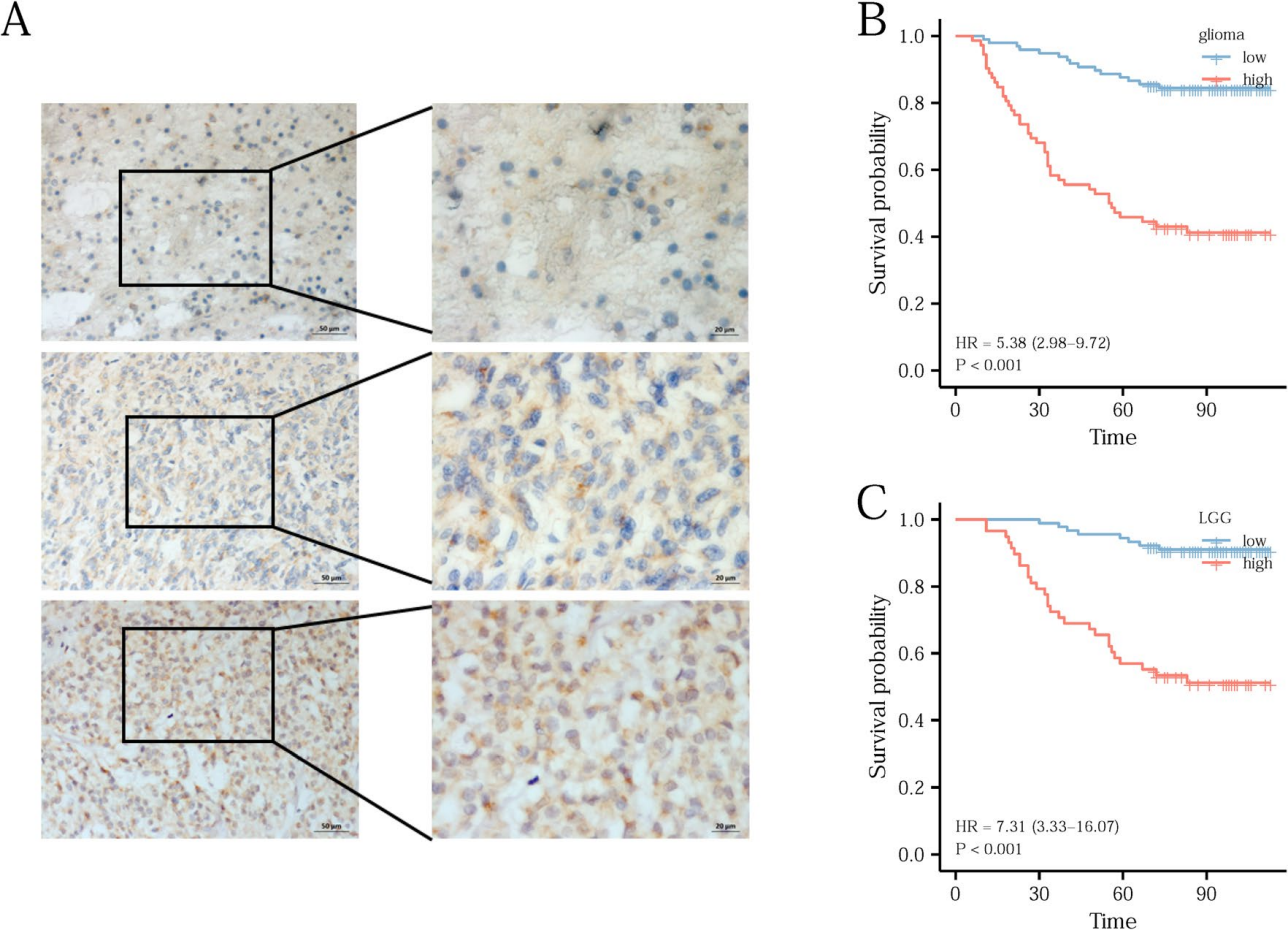
*ITGB4* expression in gliomas and its prognostic significance were analyzed by immunohistochemistry. (**A**) Shows that *ITGB4* is weakly, moderately, and strongly positive in gliomas respectively. High *ITGB4* expression was related to poor OS in glioma and lower grade glioma (**B**,**C**).

Next, we used IHC to validate the bioinformatics results and observed weak staining intensity, moderate staining, and strong staining respectively in gliomas (Fig. 4A). Tissue microarray analysis showed that overall survival (HR = 3.37, 95% CI 2.21–5.14, *p* =  < 0.001, Fig. 4B) in glioma and in LGG (HR = 6.02 95%CI 3.28–11.04), P < 0.001, Fig. 4C) was significantly poorer in cases with high *ITGB4* expression.

### High *ITGB4* expression correlated with high immune infiltration

**Figure 5:**
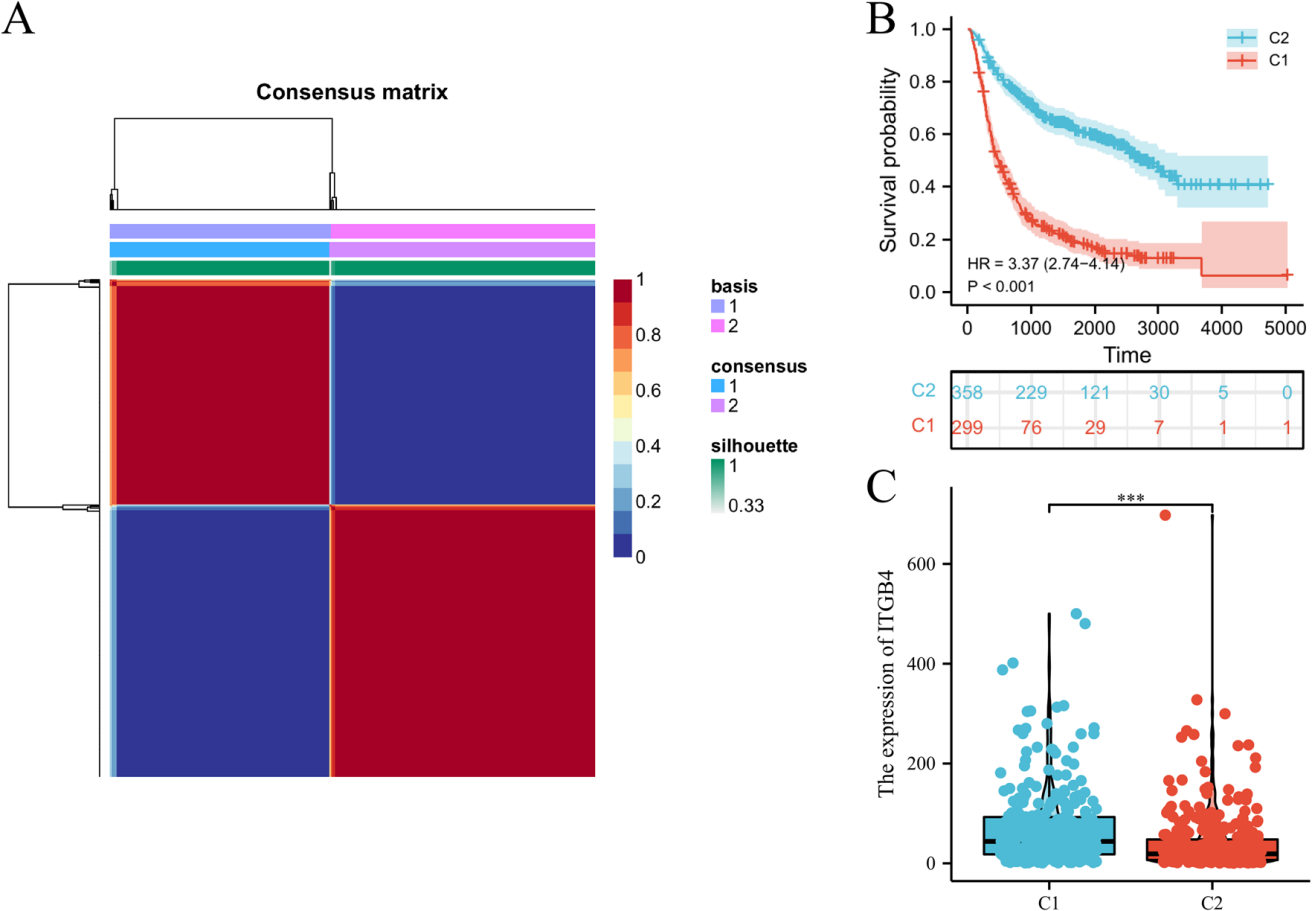
NMF cluster analysis. (**A**) According to immune related genes, glioma patients were divided into C1 and C2 groups. (**B**) Significant difference in survival between C1 and C2. (**C**) The expression of *ITGB4* was significantly different between the two groups.

**Figure 6:**
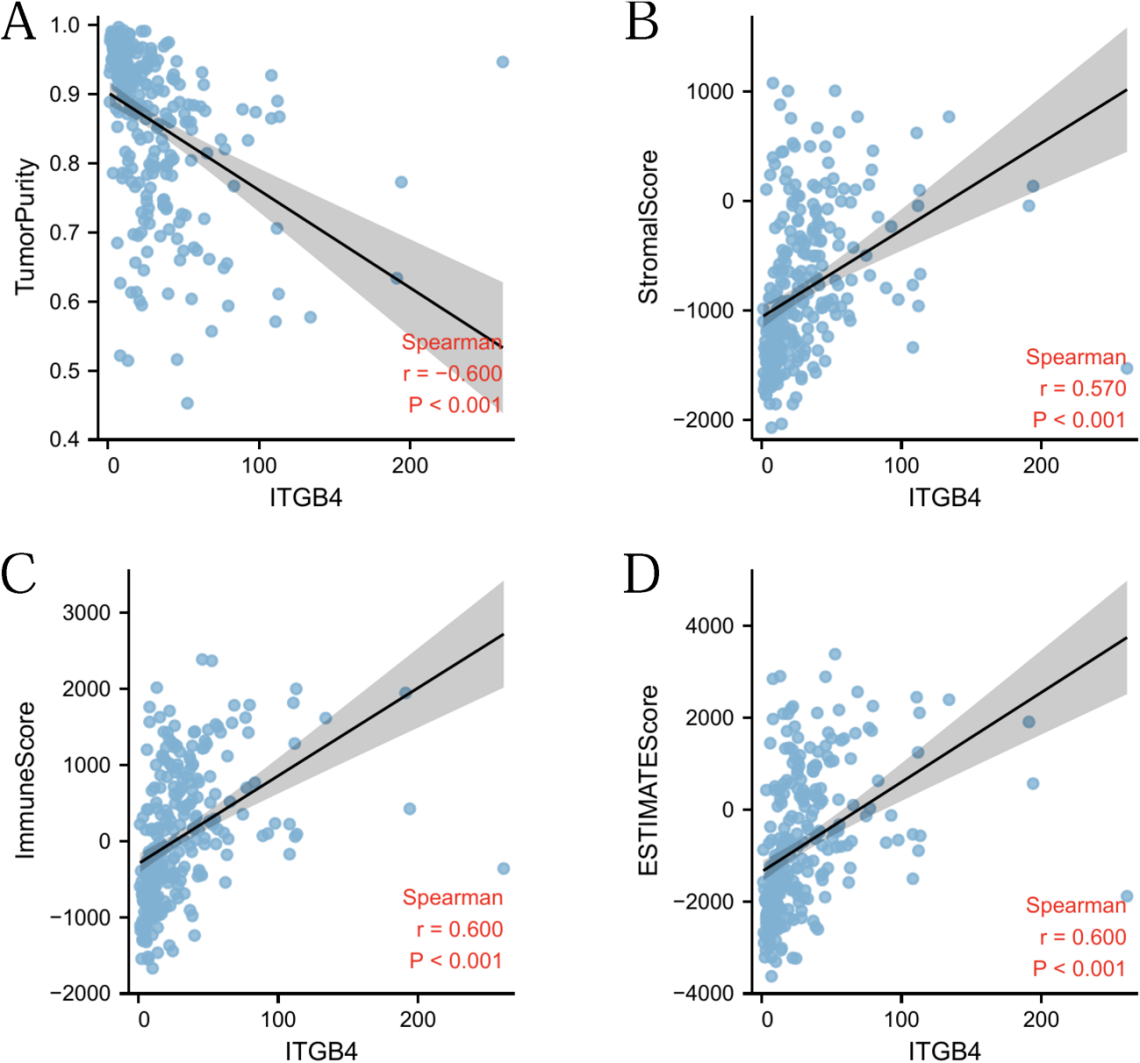
Immune infiltration patterns in CGGA lower grade glioma datasets with low and high *ITGB4* levels was analyzed using ssGSEA. (**A**–**D**) Scatter plot showing correlation between *ITGB4* and tumor purity, stromal, ESTIMATE, and immune scores.

We obtained 2,483 immune related genes from the ImmPort database, and then performed NMF clustering on glioma patients according to gene expression, survival time and survival status of patients. Through NMF cluster analysis, we divided glioma patients into two categories, C1 and C2 (Fig. 5A). Survival analysis confirmed that there was a significant difference in survival between the two groups (HR = 3.37, 95% CI 2.74–4.14, p =  < 0.001, Fig. 5B), C1 had worse survival time. Interestingly, *ITGB4* expression in C1 was significantly higher than that in C2 (Fig. 5C). Next, we used single simple gene set enrichment analysis (ssGSEA) to assess immune cell infiltration and observed that *ITGB4* expression level negatively correlated with tumor purity (r = -0.600, *p* =  < 0.001, Fig. 6A), and positively correlated with stromal score (r = 0.570, *p* =  < 0.001, Fig. 6B), ESTIMATE score (r = 0.600, P < 0.001, Fig. 6C) and immune score (r = 0.600, P < 0.001, Fig. 6D), indicating that *ITGB4* expression is closely relevant to the tumor microenvironment (TME).

### *ITGB4* functions in immune related pathways in glioma

**Figure 7:**
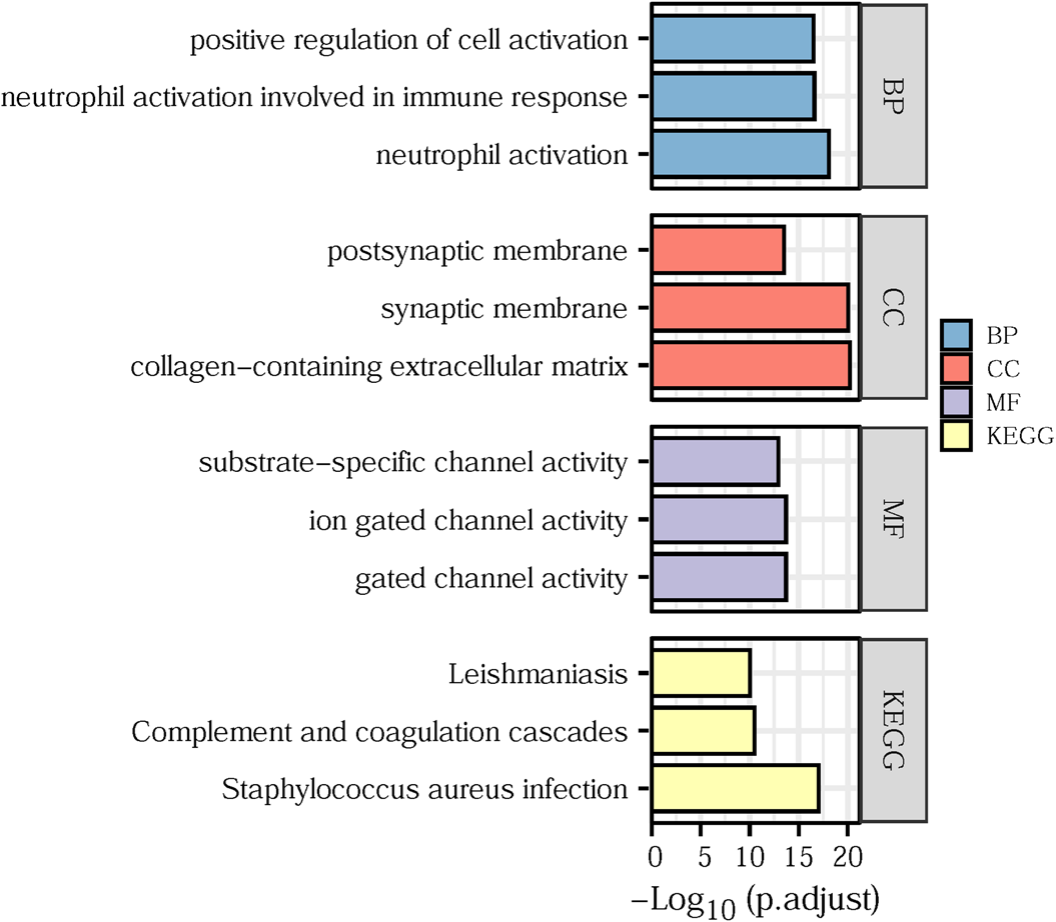
KEGG and GO analysis of the mechanism by which *ITGB4* promotes tumor progression.

Analysis of immune cell infiltration revealed that *ITGB4* is closely related to the TME in LGG. Next, investigation of the potential underlying mechanism on DAVID indicated that the DEGs were enriched in signal pathways associated with immune responses, such as neutrophil activation, collagen-containing extracellular matrix, complement and coagulation cascades, and neutrophil activation (Fig. 7). This is consistent with findings from numerous studies showing that the TME is closely associated with tumor related immunity and indicates that *ITGB4* may contribute to poor LGG prognosis via its effects on tumor immune responses.

### Single cell sequencing showed that *ITGB4* was closely related with tumor related fibroblasts

**Figure 8:**
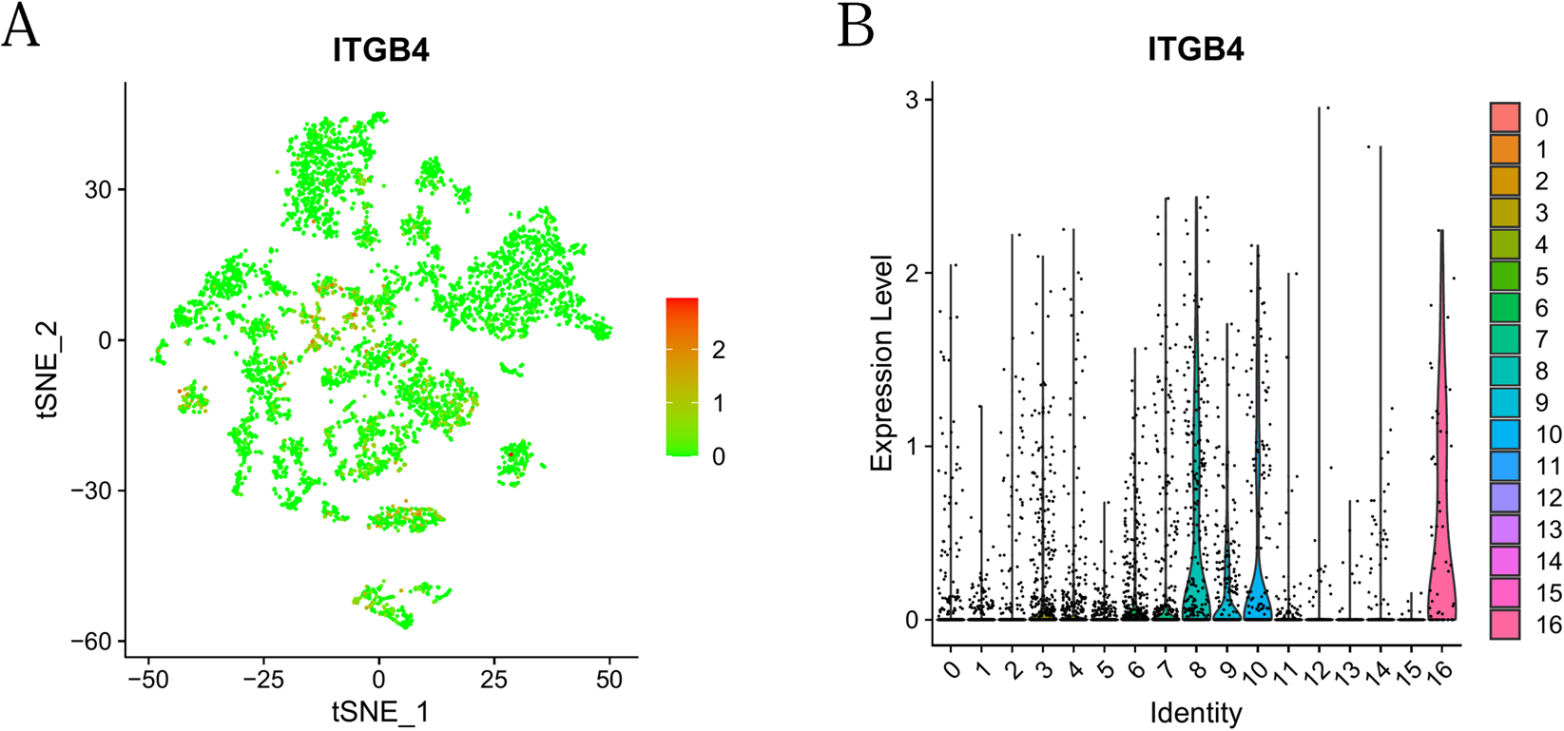
*ITGB4* expression and function in glioma were analyzed using single cell sequencing. (**A**) Scatter plot of *ITGB4* distribution in glioma. (**B**) Violin diagram of *ITGB4* distribution in the glioma microenvironment. 0 = neurons, 1 = macrophages, 2 = neurons, 3 = fibroblasts, 4 = neurons, 5 = macrophages, 6 = melanocytes, 7 = fibroblasts, 8 = fibroblasts, 9 = B cells, 10 = neurons, 11 = neurons, 12 = CD8^+^ T cells, 13 = monocytes, 14 = macrophages, 15 = macrophages, and 16 = fibroblasts.

Next, we examined *ITGB4* expression in single cell sequencing data (dataset GSE117891) obtained from GEO. Filtering and standardizing the single-cell sequencing data revealed that *ITGB4* is highly expressed in tumor-associated fibroblasts, indicating that *ITGB4* may regulate the function of fibroblasts in the TME (Fig. 8A,B).

## Discussion

Brain malignancies are estimated to account for 1.6% of new malignancies and 2.5% of annual deaths worldwide^12^. Gliomas account for 80% of brain malignancies and are mainly treated using surgery, radiation, and chemotherapy. However, glioma prognosis remains poor, especially glioblastoma which has a 5-year survival rate of 3%^13^. The poor prognosis is because glioma growth is infiltrative, difficulty in making clean glioma resections, and glioma resistance to chemoradiotherapy. Lower grade glioma (LGG) is characterized by a propensity for transformation into high-grade glioma. Thus, better understanding of glioma pathogenesis and novel diagnostic and therapeutic targets for glioma are urgently needed.

Integrins are a family of cell adhesion receptors that bind to extracellular matrix ligands, cell surface ligands, and soluble ligands. These abundant proteins participate in processes involved in the tumor biological behavior^14^. Integrins possess a large extracellular domain, a transmembrane area, and a cytoplasmic area. The extracellular domain binds to extracellular matrix (ECM) proteins like laminin while the cytoplasmic area interacts with kinases, like cytoplasmic tyrosine kinase. Its special structure and the diversity of the internal and external structures of the connected cells, confers integrin α6β4 with unique cytoskeleton and signal transduction functions. *ITGB4*, a member of the integrin family, has been implicated in tumor progression^15^. Mounting evidence indicates that *ITGB4* promotes immune escape by tumors. However, the role of *ITGB4* in glioma is poorly understood.

Here, we first performed a pan cancer analysis of *ITGB4* expression on TCGA and found that it is highly expressed in various tumors, including colorectal cancer^16^, gastric cancer^17^ and non-small lung cancer^18^. Univariate Cox regression analysis showed that *ITGB4* is strongly associated with LGG prognosis and that LGG cases with high *ITGB4* had a worse prognosis than those with lower levels. Immunohistochemical validation of the bioinformatics analysis results indicated that LGG cases with high *ITGB4* levels had worse prognosis. Similar observations have been made in some epithelial cancers. TP53 mutations and *ITGB4* upregulation co-occur in many aggressive malignancies. Squamous cell carcinomas of the lung have a higher frequency of TP53 mutations. *ITGB4* overexpression can also lead to venous invasion and reduced overall survival in non-small cell lung cancer patients^18^. *ITGB4* has also been shown to play a crucial role in the development of prostate cancer found that *ITGB4* promotes prostate tumorigenesis by co-expression with ErbB2 and c-Met in tumor progenitor cells^19^. These findings are consistent with our analysis and indicate *ITGB4* promotes tumor initiation and progression. Some studies have found that *ITGB4* expression was increased in glioma stem cells and human glioma tissues. Upregulation of *ITGB4* was correlated with glioma grades. Inhibition of *ITGB4* in glioma cells decreased the self-renewal abilities of glioma stem cells and suppressed the malignant behaviours of glioma cells in vitro and in vivo^20^. Among the ten genes selected, HLA-E, MSN, GNG-5, MYL12A, *ITGB4*, PDPN, AGTRAP, S100A4, PLSCR1, VAMP5 were selected as the most significant genes related to purity and prognosis in glioma. The risk score model based on the 10 genes could moderately predict gliomas' overall survival^21^.

Numerous studies have investigated the mechanisms by which *ITGB4* influences carcinogenesis. Recently, the tumor microenvironment (TME) has emerged as an anticancer therapeutic target. Our immune infiltration data show that *ITGB4* expression negatively correlates with tumor purity and positively correlates with immune and stromal scores, indicating that *ITGB4* may be a potential therapeutic target in the TME. Functional enrichment analysis on DAVID also indicated that *ITGB4* is closely associated with immune response. Tumor angiogenesis is an important component of TME. *ITGB4* may induce angiogenesis via the NF-κB and phospho-ERK nuclear translocation pathway, or the SRC, PI3K/Akt, and iNOS pathways^22^. Our analysis of single-cell sequencing data showed that *ITGB4* is highly expressed on tumor associated fibroblasts. Interestingly, a recent study showed that *ITGB4* mediates metabolic reprogramming of tumor associated fibroblasts^23^. These results indicate that *ITGB4* closely interacts with the TME, particularly with tumor associated fibroblasts that promote tumor development.

Our data show that *ITGB4* expression was higher in LGG cases with poor prognosis. Additionally, *ITGB4* is closely related to immune cell infiltration in the glioma TME, and specifically interacts with tumor associated fibroblasts. Taken together, our data show that *ITGB4* is a promising diagnostic and therapeutic factor for LGG. However, our findings need further in vivo and in vitro validation.

### Supplementary Information

Supplementary Information.

## Data Availability

The data analyzed in this paper were obtained from tumor-related databases. RNA sequencing data mRNAseq-693 and mRNAseq-325 and the corresponding clinical information from the CGGA were obtained for analysis (http://www.cgga.org.cn/). LGG and GBM cohorts were downloaded from TCGA data portal (https://portal.gdc.cancer.gov/).
