# Supplementary material for: ITGB4 upregulation is associated with progression of lower grade glioma
Source: Sci Rep. 2024 Jan 3;14:421. doi: 10.1038/s41598-023-49801-y (PMC10764336; doi:10.1038/s41598-023-49801-y)
Supplement: Supplementary file 1 — Supplementary Information. [file 41598_2023_49801_MOESM1_ESM.docx]

BLCA Bladder Urothelial Carcinoma

CESC Cervical squamous cell carcinoma and endocervical adenocarcinoma

CHOL Cholangiocarcinoma

COAD Colon adenocarcinoma

DLBC Lymphoid Neoplasm Diffuse Large B-cell Lymphoma

GBM Glioblastoma multiforme

HNSC Head and Neck squamous cell carcinoma

LIRC Local intramural residual cancer

LGG Brain Lower Grade Glioma

LIHC Liver hepatocellular carcinoma

LUAD Lung adenocarcinoma

LUSC Lung squamous cell carcinoma

OV Ovarian serous cystadenocarcinoma

PAAD Pancreatic adenocarcinoma

PCPG Pheochromocytoma and Paraganglioma

READ Rectum adenocarcinoma

STAD Stomach adenocarcinoma

TGCT Testicular Germ Cell Tumors

THCA Thyroid carcinoma

THYM Thymoma

UCEC Uterine Corpus Endometrial Carcinoma

ACC Adrenocortical carcinoma

BRCA Breast invasive carcinoma

KICH Kidney Chromophobe

PRAD Prostate adenocarcinoma

SARC Sarcoma

SKCM Skin Cutaneous Melanoma
